# Supplementary figures and images for: Wild Stinging Nettle (Urtica dioica L.) Leaves and Roots Chemical Composition and Phenols Extraction
Source: Plants (Basel). 2023 Jan 9;12(2):309. doi: 10.3390/plants12020309 (PMC9864842; doi:10.3390/plants12020309)

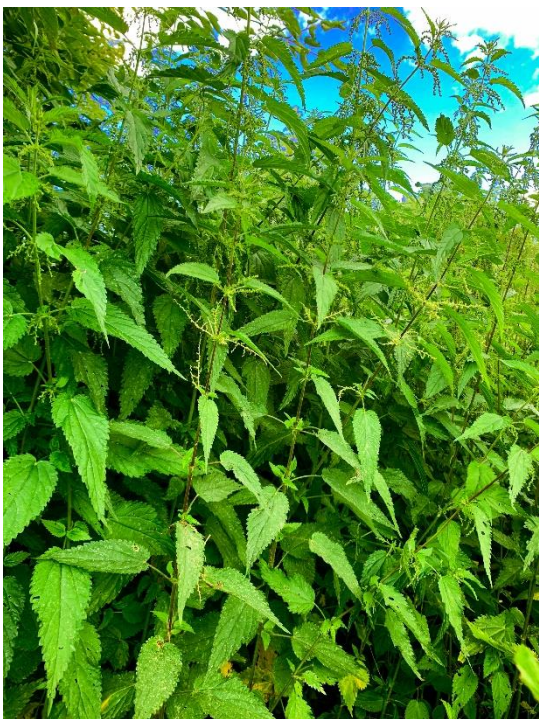

Figure S1. *Urtica dioica* L in natural environment (photo was made by Miglė Vitkauskaitė)

Supplement: Supplementary file 1 [file plants-12-00309-s001.zip › plants-2092641-supplementary.pdf]
